# Improved Translational Relevance of In Vitro Fibrosis Models by Integrating IOX2-Mediated Hypoxia-Mimicking Pathways

**DOI:** 10.3390/biomedicines13061448

**Published:** 2025-06-12

**Authors:** Manuel A. González Hernández, Jennifer Venhorst, Lars Verschuren, Karin Toet, Martien P. M. Caspers, Martine C. Morrison, Beatrice Coornaert, Gerard J. P. van Westen, Roeland Hanemaaijer

**Affiliations:** 1 Drug Discovery and Safety, Leiden Academic Centre for Drug Research, Einsteinweg 55, 2333 CC Leiden, The Netherlands; mangonzalez12@gmail.com (M.A.G.H.);; 2 Unit Healthy Living and Work, The Netherlands Organisation for Applied Scientific Research, TNO, Sylviusweg 71, 2333 BE Leiden, The Netherlandsroeland.hanemaaijer@tno.nl (R.H.); 3 Galapagos NV, Generaal de Wittelaan L11 A3, 2800 Mechelen, Belgium

**Keywords:** liver fibrosis, preclinical models, fibrosis mechanisms, hypoxia

## Abstract

**Background/Objectives**: Preclinical models of liver fibrosis only partially mimic human disease processes. Particularly, traditional transforming growth factor beta 1 (TGFβ1)-induced hepatic stellate cell (HSC) models lack relevant processes, including hypoxia-induced pathways. Here, the ability of a hypoxia-mimicking compound (IOX2) to more accurately reflect the human fibrotic phenotype on a functional level was investigated. **Methods**: Human primary HSCs were stimulated (TGFβ1 +/− IOX2), and the cell viability and fibrotic phenotype were determined. The latter was assessed as protein levels of fibrosis markers—collagen, TIMP-1, and Fibronectin. Next-generation sequencing (NGS), differential expression analyses (DESeq2), and Ingenuity Pathway Analysis (IPA) were performed for mechanistic evaluation and biological annotation. **Results**: Stimulation with TGFβ1 + IOX2 significantly increased fibrotic marker levels. Also, fibrosis-related pathways were activated, and hypoxia-related genes and collagen modifications, such as crosslinking, increased dose-dependently. Comparative analysis with human fibrotic DEGs showed improved disease representation in the HSC model in the presence of IOX2. **Conclusions**: In conclusion, the HSC model better recapitulated liver fibrosis by IOX2 administration. Therefore, hypoxia-mimicking compounds hold promise for enhancing the translational value of in vitro fibrosis models, providing valuable insights in liver fibrosis pathogenesis and potential therapeutic strategies.

## 1. Introduction

Metabolic dysfunction-associated steatotic liver disease (MASLD) and metabolic dysfunction-associated steatohepatitis (MASH) arise in the context of metabolic disease and persistent lipotoxic/glucotoxic metabolic factors in the hepatic microenvironment. In combination with pro-inflammatory factors, the transforming growth factor beta 1 (TGFβ1) canonical signaling pathway activates hepatic stellate cells (HSCs), leading to collagen deposition in the extracellular matrix (ECM) and the development of liver fibrosis [1]. Collectively, these factors trigger distinct mechanisms that increase biological heterogeneity and exacerbate disease pathogenesis. Another governing factor in liver fibrosis is hypoxia, which has been linked to both MASH progression and severity in murine models and in patients [2,3].

In humans, hypoxia, angiogenesis, and fibrogenesis synergistically contribute to liver disease progression through various mechanisms such as ECM deposition [4,5,6,7]. In addition, there is an intricate interplay between these processes since hypoxia signaling in HSCs can induce vascular endothelial growth factor (VEGF)-dependent angiogenesis in the liver [8]. In humans, several cohort studies have shown an association between sleep apnea-induced intermittent hypoxia (IH) and MASLD in obese individuals [9]. Similarly, a recent study showed that hypoxia aggravates liver fibrosis through the upregulation of HIF-1α and interleukin-6 (IL-6) in human fibrotic tissues, a CCl_4_-induced mouse model (C57BL/6J), and a human stellate cell line (LX-2), where IL-6-mediated IL-17A secretion exacerbated disease progression [10]. With respect to collagen fibril quality and overall ECM structure, hypoxia signaling may increase ECM crosslinks and thereby promote fibrosis progression [11]. Taken together, HIF-1α signaling appears to be a major factor in promoting fibrosis by regulating the transcription of fibrotic genes and through different mechanisms, such as angiogenesis, chemotaxis, and ECM crosslinks [12].

In recent decades, liver fibrosis has been rigorously investigated in in vitro models (LX-2 cells and human primary HSCs), often through TGFβ1 stimulation, which elicits a response in the HSC phenotype and a shift in gene expression towards a fibrotic phenotype [13]. However, such preclinical models only partially mimic the disease. As such, they fail to capture the complexity and biological heterogeneity of liver fibrosis, which in turn hampers the discovery of an effective drug therapy. Given the role of hypoxia in disease progression, the inclusion of this pathological process in an in vitro liver fibrosis model appears warranted when striving for a human translational model. The HIF-1α signaling pathway can be activated in vitro by hypoxia-mimicking compounds like IOX2 [14]. IOX2 is a selective inhibitor of prolyl hydroxylase-2 (PHD2), with >100-fold selectivity over other 2-oxoglutarate (2OG) dependent enzymes such as histone demethylases and FIH. However, given that PHDs are part of a large family of 2OG dependent oxygenases, inhibition of enzymes other than PHD2 by IOX2 cannot be completely ruled out [15]. HIF-1α signaling pathway activation stimulates the expression of collagen crosslinking enzymes such as lysyl oxidase (LOX), lysyl oxidase-like 2 (LOXL2), and procollagen-lysine,2-oxoglutarate 5-dioxygenase 2 (PLOD2) [14]. In support of its HIF1a-activating activity, IOX2 has shown transcriptional induction of these crosslinking enzymes in human lung fibroblasts [14].

This work aims to validate the hypothesis that 1) TGFβ1 stimulation in combination with a hypoxia-mimicking compound (IOX2) exacerbates the HSC fibrotic phenotype through fibrotic markers (Collagen, TIMP-1, Fibronectin), and collagen crosslinks (pyridinoline/collagen), and 2) in vitro mimicking HIF-1α signaling improves the translational relevance of a primary human HSC model for liver fibrosis. Once validated, the improved translational models can accelerate the drug development process for liver fibrosis treatment.

## 2. Materials and Methods

### 2.1. Cell Culture

Cell culture was performed as described before [16]. Cryopreserved primary healthy human HSCs (iXCells Biotechnologies, Lot#PFP, #25914) were thawed and plated for expansion in Poly-L-Lysine (PLL) (2 μg/cm^2^) (ScienCell Research Laboratories, Carlsbad, CA, USA) 75 cm^2^ coated flasks (Greiner, Cat#658175, Alphen aan de Rijn, The Netherlands) to enhance cell attachment. Primary HSCs were seeded in Stellate cell medium (SteCM; ScienCell Research Laboratories) containing 2% FBS 1%, penicillin/streptomycin (PS) and 1% Stellate cell growth supplement (SteCGS) (ScienceCell, SCC5301). Cells were incubated at 37 °C and 5% CO_2_. After 4 h, when cells were attached to the bottom of the flask, they were refreshed with a new SteCM medium. Cells were passaged after 7 days of culture by detaching them with 2.5 mL/flask accutase (Sigma, Cat#A6964, Amsterdam, The Netherlands). After 3–5 min incubation, accutase was inactivated with 7.5 mL/flask SteCM medium. Cells were counted with the Scepter™ 3.0 Cell Counter with 60 μm counter sensors and seeded in 24-well cell culture plates (Costar Corning, Cat#3524, Corning NY, USA) coated with fibronectin (Merck Life Science #10838039001, Amsterdam, The Netherlands) at a density of 3.69 × 10^4^ cells/well in a final volume of 500 μL/well.

The following day (day 1), before trigger solutions were added, cells were washed with SteCM containing 1% FBS and 1% PS. Trigger solutions were prepared in complete SteCM containing 1% FBS, 1% PS, 1% insulin-transferrin-sodium selenite (ITS) (Sigma, Cat#I3146), 173 μM ascorbic acid (AA) (Sigma, Cat#A8960) [17], 2.5 mM L-proline (Sigma, P5607), 2.5 mM lysine (Sigma, Cat#62840), and 2.5 mM glycine (Sigma, Cat#G5417).

Trigger solutions included combinations of TGF-β1 (R&D Systems, Cat#240-B, Abingdon, UK) at a concentration of 4 ng/mL, Alk5 inhibitor (Millipore #616459, Amsterdam, The Netherlands) at a concentration of 5 μM, and IOX2 (Sigma, Cat#SML0652) at concentrations of 0.3 μM and 1 μM. In total, 8 different trigger solutions (control, IOX2 0.3, IOX2 1, TGF-β1, TGF-β1 + Alk5, TGF-β1 + IOX2 0.3, TGF-β1 + IOX2 0.3 + Alk5 and TGF-β1 + IOX2 1) were added in a final volume of 300 μL/well and incubated for 4 days.

### 2.2. Total Collagen, Total Protein and Conditioned Media

On day 4, cells were washed with PBS, hydrolyzed (1 h) with 120 μL/well 6 M HCl and harvested for total collagen and protein quantification (Merck, Cat#1.00317.1000). Collagen and protein levels were measured using the Sensitive Tissue Collagen Assay (Quickzyme Biosciences, Cat#QZBtiscol2, Leiden, The Netherlands) and Total Protein Assay (Quickzyme Biosciences, Cat#QZBtoprot2), according to the manufacturer’s instructions. Absorbance in 96 well assay plates was measured at 570 nm using the Synergy™ HTX Multi-Mode Microplate Reader. Human fibronectin and TIMP-1 protein expression were measured in the conditioned medium collected on day 4 using ELISA (R&D Systems, Abingdon, UK).

### 2.3. Crosslinks

Samples (Control, IOX2 0.3, IOX2 1, TGF-β1, TGF-β1 + IOX2 0.3, and TGF-β1 + IOX2 1) were incubated for 6 days, HCl-hydrolyzed (120 μL/well 6 M HCl), and then incubated (37 °C and 5% CO_2_) for non-enzymatic crosslink maturation for 21 days to ensure full maturation [18]. Cell hydrolysates in triplicates were fully dried under nitrogen flow and reconstituted in elution buffer (48007, Chromsystems Instruments & Chemicals GmbH, Gräfelfing, Germany). Pyridinoline crosslinks were measured by ultra-performance liquid chromatography (ACQUITY UPLC H-Class, Waters Chromatography B.V., Etten-Leur, The Netherlands) equipped with an HPLC column (48100, Chromsystems) using an isocratic separation method (mobile phase 48001, Chromsystems; flow rate 1.2 mL/min) with subsequent fluorescent detection (EX 290 nm, EM 395 nm; ACQUITY UPLC Fluorescence Detector, Waters). Total collagen was measured in the same cell hydrolysates used for crosslink measurement using a Sensitive Tissue Collagen Assay (Quickzyme Biosciences, Cat#QZBtiscol2, Leiden, The Netherlands) according to the manufacturer’s instructions. Pyridinoline crosslinks were expressed as crosslinks/collagen (mol/mol).

### 2.4. Statistics

The primary objective was to understand the group effects rather than the interaction between groups and dosages. Therefore, for this analysis, we performed one-way ANOVA (non-parametric Kruskal–Wallis test) followed by Dunnett’s post hoc test for multiple comparisons test using GraphPad Prism version 10.0.0 for the Windows operating system (GraphPad Software, Boston, MA, USA). Statistical significance is depicted as follows: ** *p*-value < 0.01 and * *p*-value < 0.05 relative to control. # *p*-value < 0.01 relative to TGFβ1.

### 2.5. Cell Viability and Toxicity

Cell viability and cytotoxicity was determined in triplicate in the screening of the wide range of concentrations of hypoxia-mimicking compounds. CyQUANT LDH cytotoxicity assay (Invitrogen, #C20301, Bleiswijk, The Netherlands) and AlamarBlue Cell viability (Invitrogen, DAL1100) were conducted according to the manufacturer’s instructions.

### 2.6. Transcriptomics

#### 2.6.1. Data Processing

In addition, next-generation sequencing (NGS) was performed in RNA samples extracted from the 8 conditions mentioned above in biological duplicates. RNA was isolated using the RNAqeus Microkit (Thermo Fisher Scientific, Cat#AM1931, Bleiswijk, The Netherlands), and RNA concentrations were determined using NanoDrop 2000 (Unigen Life Sciences, Westburg, Leusden, The Netherlands). RNA samples were stored at −80 °C until the further sample DNA cleaning step. The NEBNext Ultra II Directional RNA library Prep Kit for Illumina (New England Biolabs, Ipswich, MA, USA) was used to process the samples, following the manufacturer’s instructions. Strand-specific messenger RNA sequencing libraries were clustered and sequenced on a NovaSeq6000 system (paired-end 20 M reads/sample, Illumina, San Diego, CA, USA) at Genomescan (Leiden, The Netherlands). Quality was checked with the Illumina data analysis pipeline RTA3.4.4 and Bcl2fastq v2.20 and filtered and trimmed using TRIMMOMATIC software (trimmomatic-0.38-1). These trimmed files were merged and aligned to the reference genome (Homo_sapiens.GRCh38.gencode.v29) with the STAR 2.5 aligner algorithm with default settings.

#### 2.6.2. Bioinformatics

Htseq-count 0.6.1p1 was used to generate the counts matrix per gene across the in vitro samples. Human count data was retrieved from a large human public dataset (216 samples) from the GEO repository (GSE135251) with a population of varying degrees of liver fibrosis (Fibrosis score 0–4) [19]. Counts matrices were used as input for differential expression analysis using DESeq2 in R to obtain differentially expressed genes (DEGs) [20]. Furthermore, DEGs (*p* < 0.01) were used as input for volcano plots and Ingenuity Pathway Analysis (IPA) (version 42012434; Ingenuity Systems; Qiagen China Co., Ltd., Shanghai, China) to examine their role in canonical pathways and directionality (z-score). Code for data preprocessing and bioinformatics analysis is provided in a git repository (https://github.com/mangonzalez12/MASLD-and-Hypoxia, 17 August 2024).

## 3. Results

**Figure 1:**
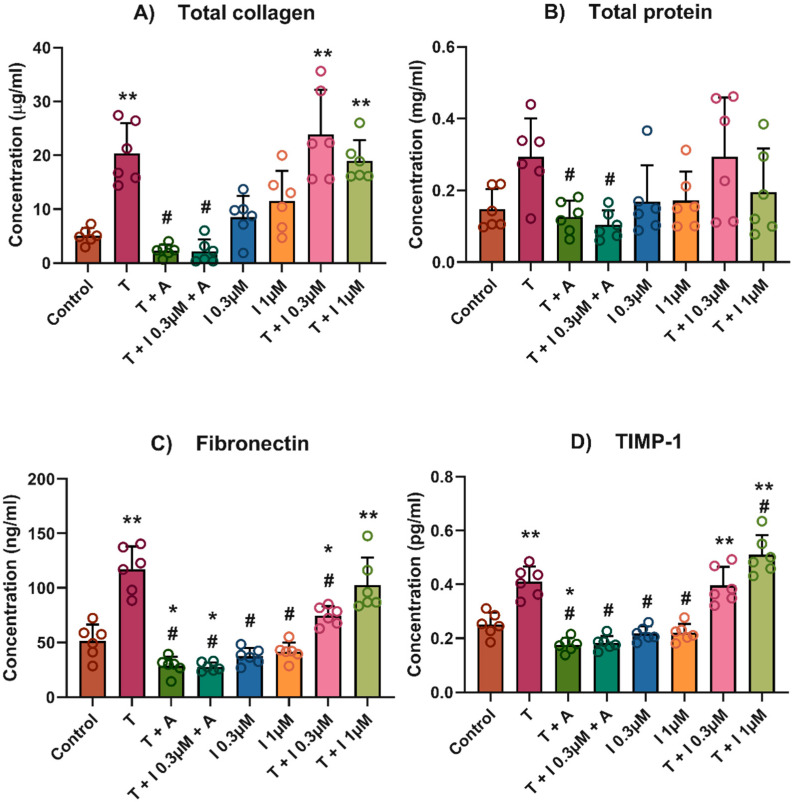
Fibrosis stimulation in primary human hepatic stellate cells with TGFβ1 and IOX2. (**A**) Total collagen, (**B**) total protein, (**C**) Fibronectin, and (**D**) TIMP-1. Trigger solutions included combinations of TGFβ1 at a concentration of 4 ng/mL, Alk5 inhibitor at a concentration of 5 μM, and IOX2 at concentrations 0.3 μM and 1 μM. In total, 8 different trigger solutions (control, IOX2 0.3 μM, IOX2 1 μM, TGFβ1, TGFβ1 + Alk5 inhibitor, TGFβ1 + IOX2 0.3 μM, TGFβ1 + IOX2 0.3 μM + Alk5 inhibitor and TGFβ1 + IOX2 1 μM) were added in a final volume of 300 μL/well and incubated for 4 days. Abbreviations: A: Alk5 inhibitor; I: IOX2, T: TGFβ1. N = 2, using triplicates. One-way ANOVA was used to compare to control and TGFβ1. ** *p*-value <0.01, * *p*-value <0.05. # *p*-value < 0.01 relative to TGFβ1.

**Figure 2:**
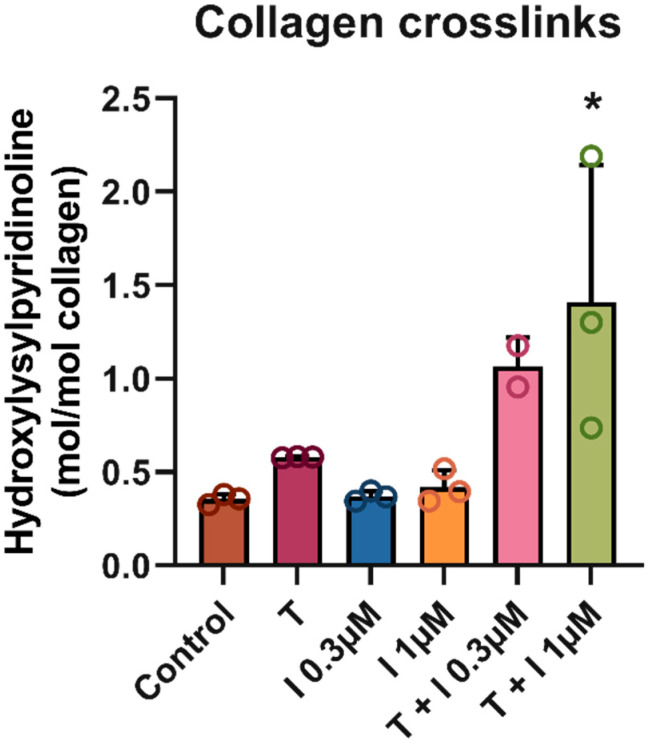
Collagen crosslinks in primary human hepatic stellate cells with TGFβ1 and IOX2. Trigger solutions included combinations of TGFβ1 at a concentration of 4 ng/mL and IOX2 at concentrations 0.3 μM and 1 μM. In total, 5 different trigger solutions (control, IOX2 0.3 μM, IOX2 1 μM, TGFβ1, TGFβ1 + IOX2 0.3 μM and TGFβ1 + IOX2 1 μM) were added in a final volume of 300 μL/well and incubated for 6 days; then, they were HCl-hydrolyzed and matured for 21 days. Abbreviations: I: IOX2, T: TGFβ1. N = 1, using triplicates. One-way ANOVA was used to compare to control. * *p*-value < 0.05.

The hypoxia-mimicking compound IOX2 was assessed for its ability to enhance the TGFβ1-induced fibrotic phenotype in human primary hepatic stellate cells (HSCs) at multiple concentrations. Stimulation of cultured HSCs with TGFβ1 in the presence/absence of IOX2 (0.3 and 1 µM) resulted in a significant increase in total collagen, TIMP-1, and fibronectin relative to the non-stimulated control (*p*-value < 0.01) (Figure 1). Of note, the addition of an ALK5 inhibitor (5 µM) fully inhibited the HSC stimulation induced by TGFβ1 and TGFβ1 + IOX2 0.3 µM (*p*-value < 0.01). IOX2 (0.3, 1 µM) alone showed a gradual increase in total collagen levels in a dose-dependent manner. Interestingly, TIMP-1 concentrations were also significantly higher in TGFβ1 + 1 µM IOX2 relative to TGFβ1 alone (*p*-value < 0.01). Compared to TGFβ1 alone, no increase was observed in total collagen and fibronectin in cells treated with TGFβ1 + 1 μM IOX2. However, analysis of mature collagen crosslinks using different conditions (Figure 2) showed that the ratio of pyridinoline crosslinks/collagen was significantly higher upon addition of IOX2 compared with the TGFb1-stimulated control (*p*-value < 0.05, Figure 2).

Using the experimental conditions described above, the effect of IOX2 was next comprehensively assessed in our HSC model on the gene and pathway levels. To this end, NGS was performed with the objective to investigate activation of the HIF-1α signaling pathway and overall fibrosis-related mechanisms using Ingenuity Pathway Analysis.

**Figure 3:**
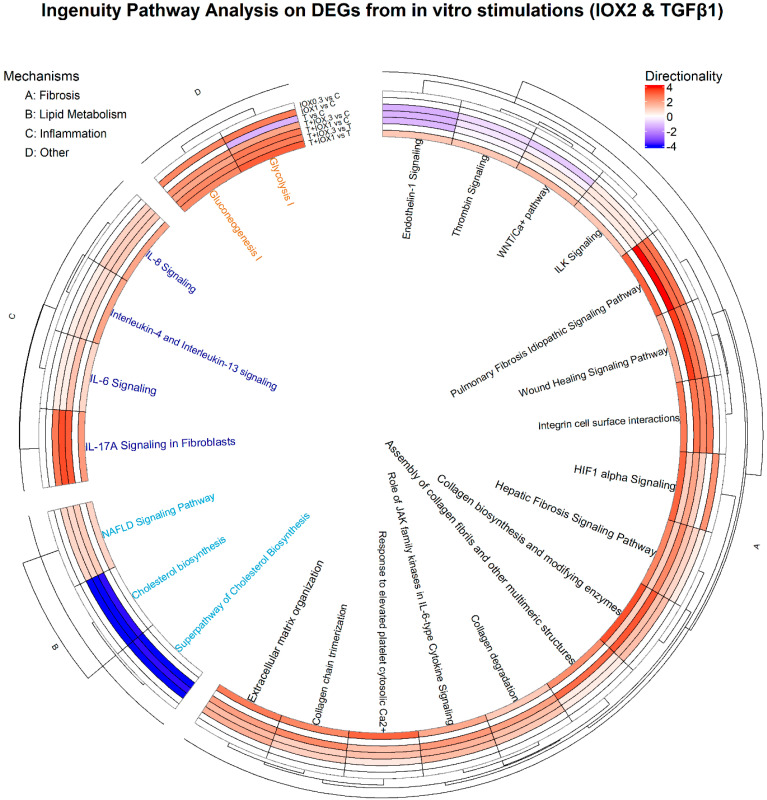
Predicted activation states of canonical pathways (z-score) in primary human hepatic stellate cells stimulated with TGFβ1 and/or IOX2. Differentially expressed genes from different pairwise comparisons (IOX2 0.3 μM, IOX2 1 μM, TGFβ1, TGFβ1 + IOX2 0.3 μM and TGFβ1 + IOX2 1 μM) relative to control and TGFβ1 + IOX2 0.3 and TGFβ1 + IOX2 1 relative to TGFβ1 were used for IPA. Values represent the directionality (z-score), which shows the predicted activation state of fibrosis-related lipid metabolism, inflammation, and other pathways.

Pairwise comparisons of DEGs from IOX2, TGFβ1, or co-treated HSCs relative to their respective controls (*p*-value < 0.01) demonstrated that the combination of TGFβ1 with 1 µM IOX2 exacerbates the activation of HIF-1α signaling pathway, as indicated by increased scores (z-score) at this dose of IOX2 with TGFβ1 (Figure 3). Furthermore, various disease mechanisms, including fibrosis-related mechanisms (i.e., wound healing signaling pathway, hepatic fibrosis signaling pathway, collagen biosynthesis, and enzyme modification), as well as inflammation pathways (i.e., Il-6 signaling, Il-17 signaling), showed a gradual increase with the addition of IOX2 in a concentration-dependent manner.

**Figure 4:**
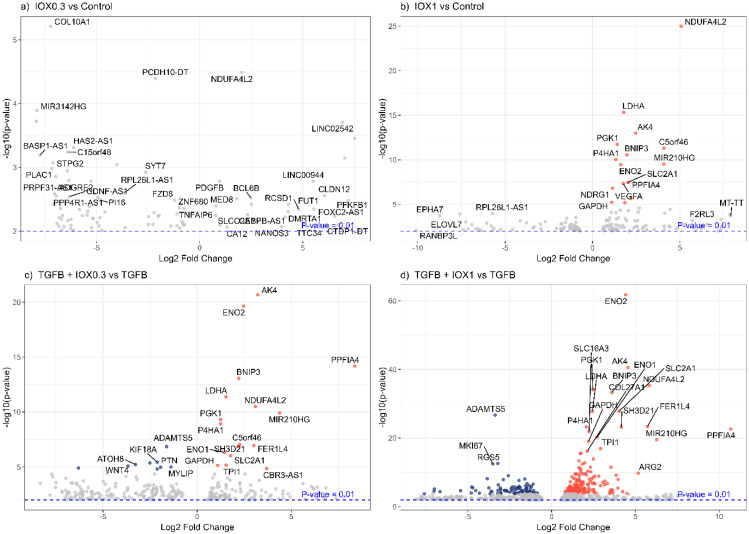
Hypoxia-relevant genes differentially expressed in various conditions. (**a**) IOX2 0.3 μM and (**b**) IOX2 1 μM relative to control and (**c**) (TGFβ1 + 0.3 μM) and (**d**) (TGFβ1 + 1 μM) relative to TGFβ1. DEGs (*p* < 0.01) were used as input for volcano plots.

To investigate whether IOX2 can elicit a response on hypoxia-relevant genes, DEGs were visualized in volcano plots in different pairwise comparisons (Figure 4). Of note, various DEGs showing highest significance (i.e., *ENO1*, *ENO2*, *LDHA*, *GAPDH*, *BNIP3*, *PDK1*, *TGFBI*) are present in the Hypoxia Hallmark gene set from GSEA (Gene Set Enrichment Analysis) [21]. Collectively, these data suggest that the addition of IOX2 to an in vitro model of liver fibrosis, i.e., TGFβ1-induced HSC activation, simulates the hypoxic processes that play an important role in liver fibrosis, as evidenced by the induction of hypoxia-relevant genes and the observed aggravated disease phenotype. Consequently, IOX2 addition more comprehensively captures the disease processes at play in MASH and MASLD, improving the translational value of the in vitro model.

To investigate the relevance of our human primary HSC model in the context of whole-liver human in vivo complexity, DEGs from a human transcriptome dataset derived from MASH patients (F4 vs. F0 fibrosis score) were compared to those derived from our in vitro experiments. Interestingly, a gradual increase in the number of DEGs shared between the in vitro and the clinical datasets was observed when in vitro IOX2 was added, indicating improved recapitulation of human disease processes (Table 1). In concurrence, higher gene expression of various crosslinking core enzymes (i.e., *PLOD1*, *PLOD2*, *LOX*, *LOXL3*) was observed in samples treated with IOX2 alone, whereas IOX2 synergistically increased the effect of TGFβ1 on the gene expression of these enzymes (Supplementary Figure S1).

## 4. Discussion

Metabolic Dysfunction-Associated Steatohepatitis (MASH) is the consequence of the intricate interplay of distinct mechanisms that manifest in a chronic manner, ultimately activating HSCs and marking the onset of liver fibrosis [22,23,24]. Although current in vitro models mimic specific aspects of pathophysiology, several of the mechanisms governing liver fibrosis are lacking, such as hypoxia. Therefore, we hypothesized that the incorporation of hypoxia signaling in these models may further increase their translational power due to a closer resemblance of the human situation in terms of mechanistic representation and fibrotic markers. To support this hypothesis, we investigated the effects of the hypoxia-mimicking compound IOX2, alongside TGFβ1 in terms of their synergistic capacity to recapitulate in vivo mechanisms in MASH patients. Analyses on the gene and protein level showed that IOX2 addition to TGFβ-induced HSCs indeed increased fibrotic markers and improved the mechanistic representation of MASH. As such, in vitro models benefit from the inclusion of hypoxic processes in terms of their translational value both for assessing the therapeutic merit of interventions and for identifying candidate drug targets, e.g., those transducing hypoxic signals.

In an in vitro study using human lung fibroblasts, stimulation with hypoxia-mimicking compounds such as IOX2 was reported to induce crosslinking enzymes at the gene expression level (i.e., *PLOD2*, *LOXL2*) [14]. We extended this observation to human liver stellate cells (HSCs), and in more detail examined the effect on fibrosis-related crosslinking at the protein level and whether the hypoxia-mimicking IOX2 could better recapitulate the disease pathways present in MASH patients. In our study of HSCs, effects similar to those in lung fibroblasts were observed; IOX2-mediated hypoxia mimicking was found to enhance the expression of both fibrosis markers and crosslinking enzymes (i.e., *PLOD2*, *LOX*, *LOXL2*, *LOXL3*) in a concentration-dependent manner in the presence of TGFβ1 (See Supplementary Figure S1). Moreover, this translated not only into a shift in collagen quantity but also into a change in the collagen quality at the protein level, i.e., an increase in pyridinoline crosslinks (Figure 2), which is known to be a key factor in fibrosis progression [25]. In contrast with the study of lung fibroblasts, which used high concentrations of the hypoxia mimicking inducers (IOX2 50–250 µM) [14], much lower concentrations of IOX2 (0.3, 1 µM) were effective in our study. At higher concentrations, pronounced reduction in cell density and viability with increases in overall toxicity were observed. In further support of the role of hypoxia in liver fibrosis, both animal and human data have indicated a link between hypoxia and MASH severity [2,3]. For instance, a murine model reported that intermittent hypoxia (IH) augmented collagen levels in a time-dependent manner, thus linking hypoxia to the degree of liver fibrosis [2]. Similarly, our HSC model showed augmented collagen levels and collagen crosslinks with the addition of IOX2 on Day 4 and Day 21, respectively. To verify that the effects were due to hypoxia induction, we focused not on HIF-1α protein expression but on the molecular pathways induced by HIF-1α activation. In terms of molecular mechanisms, activation of HIF-1α signaling, as well as other related mechanisms (i.e., IL-17 signaling, BMP-1 signaling, IL-6 signaling), was observed when IOX2 was added. This is in line with a recent study reporting an upregulation of HIF-1α, IL-6, and IL-17 expression in explanted liver tissue from patients undergoing liver transplantation, as well as in a hypoxia-mimicking compound (DMOG)-treated (20 mg/kg) mouse model (C57BL/6J) [10]. Our study furthermore indicates that a central angiogenesis pathway, VEGF signaling, is exacerbated in response to hypoxia signaling (Supplementary Figure S2). This coincides with previous findings that show that hypoxia leads to VEGF-dependent angiogenesis [8]. Glycolysis is another important mechanism for the proliferation and growth of HSCs during pro-fibrotic processes. Activation of this process was also observed in the presence of IOX2 (Figure 2). This effect has previously been reported in activated HSC-LX2 cells upon manipulation of the HIF-1α signaling pathway [26].

To assess whether our in vitro model better recapitulates in vivo complexity by the addition of IOX2, we compared DEGs identified in our in vitro studies to those derived from a human whole-liver bulk-RNA-seq transcriptomics dataset (GEO repository; GSE135251). This comparison revealed a dose-dependent gradual increase in overlapping DEGs with human DEGs (F4 vs. F0) upon IOX2 addition (Table 1). In addition, various DEGs were not observed in TGFβ1-stimulated controls, indicating that they are specifically linked to IOX2 stimulation and that IOX2 elicits a unique mechanistic pattern (Table 1). As hypoxia is observed in various types of fibrosis, such as lung fibrosis and renal fibrosis and in tumor microenvironments [26], and is a clinically relevant target [27], hypoxia mimicking by IOX2 addition may also—in part—improve in vitro patient recapitulation in models outside of liver fibrosis and as such may improve future drug discovery.

## 5. Conclusions

Overall, our results show that the addition of a hypoxia-mimicking compound at low concentrations to primary human HSCs improves the mechanistic representation of pathological processes in an in vitro MASH model. This improvement is not limited to an increase in collagen expression/deposition and collagen crosslinks. Activation of other relevant fibrosis-related pathways, e.g., immune pathways, was also observed. Comparison with human data furthermore suggests that the inclusion of hypoxic processes in the in vitro HSC model may better reflect in vivo human liver fibrosis severity. These findings hold promise for the development of targeted therapeutic interventions aimed at mitigating the impact of hypoxia on liver fibrosis progression, possibly in advanced stages.

## Figures and Tables

**Table 1:** Overlap between the number of differentially expressed genes (#DEGs) observed in vitro conditions and in human MASH samples (F4 vs. F0). DEGs obtained with various in vitro conditions were compared to DEGs in a human transcriptome dataset (Fibrosis stage 4 (F4) vs. Fibrosis stage 0 (F0) (bulk-RNA seq)). The overlap is expressed as an absolute number and as a percentage of genes for up- and downregulated genes, as well as for the total number of genes. DEGs were selected with *p*-value < 0.01. T = trigger TGFβ (4 ng/mL); C = non-stimulated control; I = IOX2 concentration in μM; A = Alk-5 inhibitor (5 μM).

Modulation	Human Dataset #DEGs	In Vitro Experimental Conditions #DEGs			
	F4 vs. F0	T vs. C	T + I 0.3 vs. C	T + I 1 vs. C	T + A vs. C
Upregulated	2053	178 (8.6%)	211 (10.27%)	289 (14%)	24 (1%)
Downregulated	1217	125 (10.2%)	135 (11%)	164 (13.4%)	22 (1.8%)
Total DEGs	3270	303 (9.2%)	346 (10.6%)	453 (14%)	46 (1.4%)
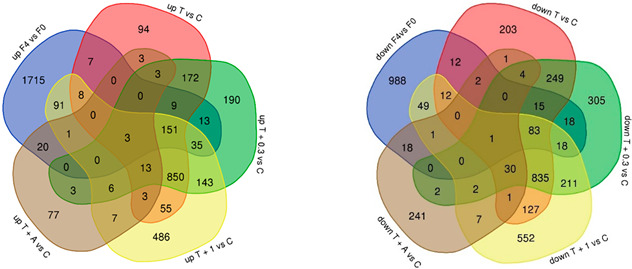					

## Data Availability

The dataset generated and analyzed during the current study is available at Gene Expression omnibus (GEO), study GSE278541, at https://www.ncbi.nlm.nih.gov/geo/ (accessed on 2 October 2024) and are available from the corresponding author on reasonable request.

## Supplementary Materials

The following supporting information can be downloaded at https://www.mdpi.com/article/10.3390/biomedicines13061448/s1; Figure S1: Crosslinking markers in distinct conditions; Figure S2: Canonical pathways directionality (z-score) in primary human hepatic stellate cells with TGFβ1 and IOX2 in TOP100 pathways.

## Abbreviations

The following abbreviations are used in this manuscript:

BMP-1	Bone Morphogenetic Protein 1
DEGs	Differentially Expressed Genes
DMOG	Dimethyloxallyl Glycine
ECM	Extracellular Matrix
EMT	Endothelial-to-Mesenchymal Transition
HCC	Hepatocellular Carcinoma
HIF-1α	Hypoxia-Inducible Factor 1 Alpha
HSC	Hepatic Stellate Cell
IH	Intermittent Hypoxia
IL-6	Interleukin-6
IL-17	Interleukin-17
IPA	Ingenuity Pathway Analysis
IOX2	1-benzyl-4-hydroxy-2-oxo-1,2-dihydroquinoline-3-carbonyl)glycine
LOX	Lysyl Oxidase
LOXL2	Lysyl Oxidase-Like 2
LOXL3	Lysyl Oxidase-Like 3
MASH	Metabolic Dysfunction-Associated Steatohepatitis
MASLD	Metabolic Dysfunction-Associated Steatotic Liver Disease
NGS	Next Generation Sequencing
PLOD2	Procollagen-Lysine, 2-Oxoglutarate 5-Dioxygenase 2
RNA-seq	RNA Sequencing
ROS	Reactive Oxygen Species
TGFβ1	Transforming Growth Factor Beta 1
VEGF	Vascular Endothelial Growth Factor

## References

[1] Kisseleva T., Brenner D. (2021). Molecular and cellular mechanisms of liver fibrosis and its regression. Nat. Rev. Gastroenterol. Hepatol..

[2] Barnes L.A., Xu Y., Sanchez-Azofra A., Moya E.A., Zhang M.P., Crotty Alexander L.E., Malhotra A., Mesarwi O. (2023). Duration of intermittent hypoxia impacts metabolic outcomes and severity of murine NAFLD. Front. Sleep.

[3] Gaucher J., Montellier E., Vial G., Chuffart F., Guellerin M., Bouyon S., Lemarie E., Botté Y.Y., Dirani A., Ben Messaoud R. (2024). Long-term intermittent hypoxia in mice induces inflammatory pathways implicated in sleep apnea and steatohepatitis in humans. iScience.

[4] Paternostro C., David E., Novo E., Parola M. (2010). Hypoxia, angiogenesis and liver fibrogenesis in the progression of chronic liver diseases. World J. Gastroenterol..

[5] Foglia B., Novo E., Protopapa F., Maggiora M., Bocca C., Cannito S., Parola M. (2021). Hypoxia, Hypoxia-Inducible Factors and Liver Fibrosis. Cells.

[6] Jia W., Liang S., Lin W., Li S., Yuan J., Jin M., Nie S., Zhang Y., Zhai X., Zhou L. (2023). Hypoxia-induced exosomes facilitate lung pre-metastatic niche formation in hepatocellular carcinoma through the miR-4508-RFX1-IL17A-p38 MAPK-NF-κB pathway. Int. J. Biol. Sci..

[7] Strowitzki M.J., Kirchberg J., Tuffs C., Schiedeck M., Ritter A.S., Biller M., Harnoss J.M., Lasitschka F., Schmidt T., Radhakrishnan P. (2018). Loss of Prolyl-Hydroxylase 1 Protects against Biliary Fibrosis via Attenuated Activation of Hepatic Stellate Cells. Am. J. Pathol..

[8] Dirscherl K., Schläpfer M., Roth Z’graggen B., Wenger R.H., Booy C., Flury-Frei R., Fatzer R., Aloman C., Bartosch B., Parent R. (2020). Hypoxia sensing by hepatic stellate cells leads to VEGF-dependent angiogenesis and may contribute to accelerated liver regeneration. Sci. Rep..

[9] Minville Dr C., Hilleret Dr M.N., Tamisier R., Aron-Wisnewsky J., Clement K., Trocme C., Borel J.C., Lévy P., Zarski Dr J.P., Pépin J.L. (2014). Nonalcoholic fatty liver disease, nocturnal hypoxia, and endothelial function in patients with sleep apnea. Chest.

[10] Kou K., Li S., Qiu W., Fan Z., Li M., Lv G. (2023). Hypoxia-inducible factor 1α/IL-6 axis in activated hepatic stellate cells aggravates liver fibrosis. Biochem. Biophys. Res. Commun..

[11] Lloyd S.M., He Y. (2024). Exploring Extracellular Matrix Crosslinking as a Therapeutic Approach to Fibrosis. Cells.

[12] Roth K.J., Copple B.L. (2015). Role of Hypoxia-Inducible Factors in the Development of Liver Fibrosis. Cell. Mol. Gastroenterol. Hepatol.—CMGH.

[13] Dewidar B., Meyer C., Dooley S., Meindl-Beinker N. (2019). Tgf-β in hepatic stellate cell activation and liver fibrogenesis—Updated 2019. Cells.

[14] Brereton C.J., Yao L., Davies E.R., Zhou Y., Vukmirovic M., Bell J.A., Wang S., Ridley R.A., Dean L.S.N., Andriotis O.G. (2022). Pseudohypoxic HIF pathway activation dysregulates collagen structure-function in human lung fibrosis. Elife.

[15] Chan M.C., Atasoylu O., Hodson E., Tumber A., Leung I.K., Chowdhury R., Gómez-Pérez V., Demetriades M., Rydzik A.M., Holt-Martyn J. (2015). Potent and Selective Triazole-Based Inhibitors of the Hypoxia-Inducible Factor Prolyl-Hydroxylases with Activity in the Murine Brain. PLoS ONE.

[16] Gart E., van Duyvenvoorde W., Toet K., Caspers M.P.M., Verschuren L., Nielsen M.J., Leeming D.J., Souto Lima E., Menke A., Hanemaaijer R. (2021). Butyrate Protects against Diet-Induced NASH and Liver Fibrosis and Suppresses Specific Non-Canonical TGF-β Signaling Pathways in Human Hepatic Stellate Cells. Biomedicines.

[17] Smith-Cortinez N., Fagundes R.R., Gomez V., Kong D., de Waart D.R., Heegsma J., Sydor S., Olinga P., de Meijer V.E., Taylor C.T. (2021). Collagen release by human hepatic stellate cells requires vitamin C and is efficiently blocked by hydroxylase inhibition. FASEB J..

[18] Van der Slot-Verhoeven A.J., van Dura E.A., Attema J., Blauw B., Degroot J., Huizinga T.W., Zuurmond A.M., Bank R.A. (2005). The type of collagen cross-link determines the reversibility of experimental skin fibrosis. Biochim. Biophys. Acta.

[19] Govaere O., Cockell S., Tiniakos D., Queen R., Younes R., Vacca M., Alexander L., Ravaioli F., Palmer J., Petta S. (2020). Transcriptomic profiling across the nonalcoholic fatty liver disease spectrum reveals gene signatures for steatohepatitis and fibrosis. Sci. Transl. Med..

[20] Love M.I., Huber W., Anders S. (2014). Moderated estimation of fold change and dispersion for RNA-seq data with DESeq2. Genome Biol..

[21] Subramanian A., Tamayo P., Mootha V.K., Mukherjee S., Ebert B.L., Gillette M.A., Paulovich A., Pomeroy S.L., Golub T.R., Lander E.S. (2005). Gene set Enrichment Analysis: A Knowledge-Based Approach for Interpreting Genome-Wide Expression Profiles. Proc. Natl. Acad. Sci. USA.

[22] Kisseleva T., Ganguly S., Murad R., Wang A., Brenner D.A. (2025). Regulation of Hepatic Stellate Cell Phenotypes in MASH. Gastroenterology.

[23] Cai J., Hu M., Chen Z., Ling Z. (2021). The roles and mechanisms of hypoxia in liver fibrosis. J. Transl. Med..

[24] French S.W. (2004). The role of hypoxia in the pathogenesis of alcoholic liver disease. Hepatol. Res..

[25] Wang F., Chen L., Kong D., Zhang X., Xia S., Liang B., Li Y., Zhou Y., Zhang Z., Shao J. (2024). Canonical Wnt signaling promotes HSC glycolysis and liver fibrosis through an LDH-A/HIF-1α transcriptional complex. Hepatology.

[26] Mallikarjuna P., Zhou Y., Landström M. (2022). The Synergistic Cooperation between TGF-β and Hypoxia in Cancer and Fibrosis. Biomolecules.

[27] Papadopoulou D., Mavrikaki V., Charalampous F., Tzaferis C., Samiotaki M., Papavasileiou K.D., Afantitis A., Karagianni N., Denis M.C., Sanchez J. (2024). Discovery of the First-in-Class Inhibitors of Hypoxia Up-Regulated Protein 1 (HYOU1) Suppressing Pathogenic Fibroblast Activation. Angew. Chem. Int. Ed. Engl..

