# Supplementary material for: Improved Translational Relevance of In Vitro Fibrosis Models by Integrating IOX2-Mediated Hypoxia-Mimicking Pathways
Source: Biomedicines. 2025 Jun 12;13(6):1448. doi: 10.3390/biomedicines13061448 (PMC12190251; doi:10.3390/biomedicines13061448)
Supplement: Supplementary file 1 [file biomedicines-13-01448-s001.zip › biomedicines-3540068-supplementary.pdf]

Supplementary material

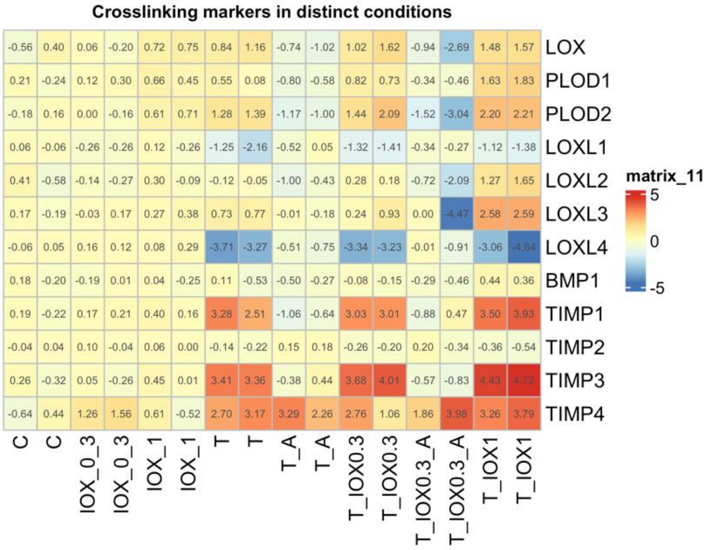

Supplementary Figure S1. Expression of crosslinking markers in distinct conditions. Increased expression upon IOX2 addition in both the presence and absence of TGFβ1 is observed for LOX, PLOD1, PLOD2 and LOXL3. Trigger solutions included combinations of IOX2 at concentrations 0.3 and 1 μM in the presence/absence of TGFβ1 (4 ng/ml). Alk-5 inhibitor (5 μM) was used as positive control for TGFβ1 signaling inhibition. The following conditions were used: control, IOX2 0.3 μM, IOX2 1 μM, TGFβ1, TGFβ1 + Alk-5, TGFβ1 + IOX2 0.3 μM, TGFβ1 + IOX2 0.3 μM + Alk-5, and TGFβ1 + IOX2 1 μM). 2logr transformation was applied correcting for control group.

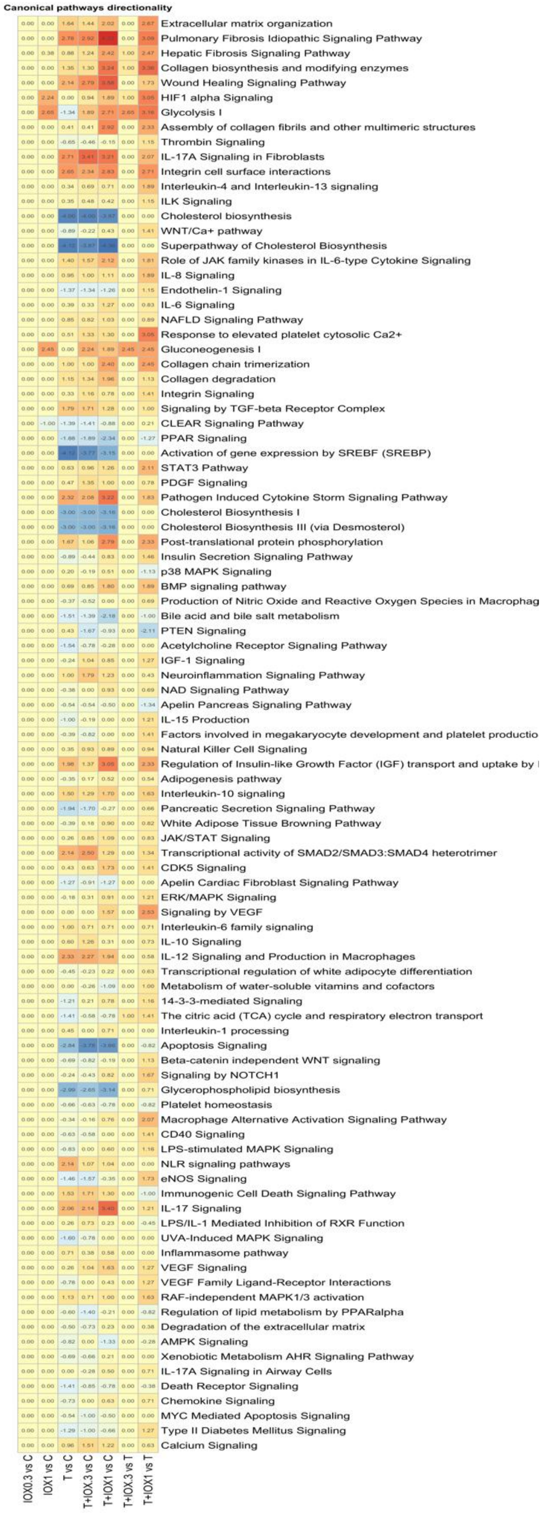

Supplementary Figure S2. Canonical pathways directionality (z-score) in primary human hepatic stellate cells with TGFβ1 and IOX2 in TOP100 pathways. Differentially expressed genes from different pairwise comparisons (IOX2 0.3, IOX2 1, TGFβ1, TGFβ1 + IOX2 0.3 and TGFβ1 + IOX2 1) relative to control and TGFβ1 + IOX2 0.3 and TGFβ1 + IOX2 1 relative to TGFβ1 were used for IPA analysis. Values represent the

directionality (z-score) which show up and downregulation of fibrosis-related, lipid metabolism, inflammation, and other pathways.
